# A Review of the Role of the Gut Microbiome in Personalized Sports Nutrition

**DOI:** 10.3389/fnut.2019.00191

**Published:** 2020-01-10

**Authors:** Riley L. Hughes

**Affiliations:** Department of Nutrition, University of California, Davis, Davis, CA, United States

**Keywords:** gut microbiome, exercise, personalized nutrition, sports nutrition, performance, metabolism, athletes, optimization

## Abstract

The gut microbiome is a key factor in determining inter-individual variability in response to diet. Thus, far, research in this area has focused on metabolic health outcomes such as obesity and type 2 diabetes. However, understanding the role of the gut microbiome in determining response to diet may also lead to improved personalization of sports nutrition for athletic performance. The gut microbiome has been shown to modify the effect of both diet and exercise, making it relevant to the athlete's pursuit of optimal performance. This area of research can benefit from recent developments in the general field of personalized nutrition and has the potential to expand our knowledge of the nexus between the gut microbiome, lifestyle, and individual physiology.

## Introduction

The gut microbiome has been implicated in the modulation of human health and metabolism (1, 2). This microbial “organ” has been linked to nutrition-related chronic diseases such as obesity and diabetes (3–6) and has also been shown to influence systemic functions including immunity (7, 8) and brain function (9, 10). The gut microbiome may influence health via mechanisms such as the production of metabolites (2, 11) [e.g., short-chain fatty acids (SCFAs)] that can influence a wide array of host systems and metabolic pathways (12, 13).

**Figure 1:**
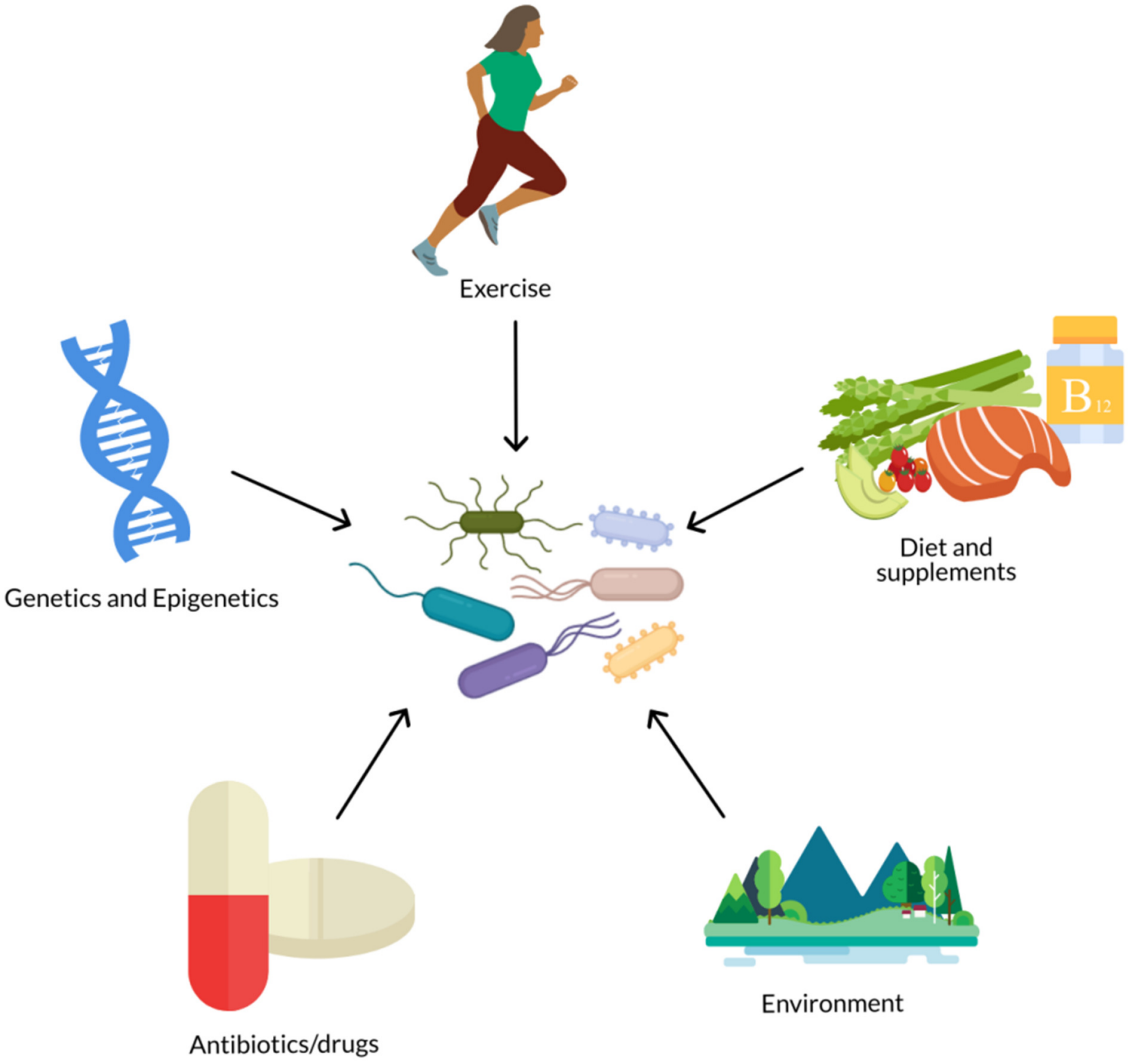
The gut microbiome is influenced by numerous biological and lifestyle factors such as diet, genetics, antibiotics, exercise, and environment (e.g., pollutants, urban vs. rural, etc.).

However, the gut microbiome is not a fixed trait, but instead responds to environmental stimuli and is a malleable part of the human supraorganism (14) (Figure 1). Much of microbiome research has focused on the effect of lifestyle factors, such as diet (15–17) and exercise (18, 19), on the gut microbiota. Variability in the composition and function of the gut microbiome (20, 21) has also fueled research on the relationship between features of the gut microbiota, such as diversity or the presence, absence, or amount of certain taxa, and host health. Precision nutrition studies are now investigating how to predict individual differences in glycemic response, triglycerides, cholesterol levels, and other indicators of health as a way to personalize nutrition recommendations and prevent diet-related chronic diseases such as obesity and type 2 diabetes. Our previous two-part review (22, 23) explored the effect of the gut microbiome on inter-individual variability in response to diet and how this may contribute to metabolic health.

Alternatively, we may consider the potential effect of the gut microbiome on measures of athletic performance. Successful performance in training, such as a time trial or rep max load, and general metabolic health are two distinct aspects of metabolic response that are not necessarily directly coupled (24). While nutrition is an important part of general health and well-being, it is also an important tool in an athlete's arsenal to optimize performance (25).

Variability in the physiological response to training and nutrition has been attributed to factors such as age, sex, training history, initial training status, psychological factors, and the mode, duration, intensity, and frequency of training (26). Genetics has also become a large topic of research in the area of variability in response to exercise training and potentially ergogenic dietary components (27–38). It is possible that variability in the gut microbiome may also influence gains in performance in response to training and nutrition. Despite the growing interest in the gut microbiome and personalized nutrition, very few studies have combined these fields with that of athletic performance. This is surprising as athletes are extremely motivated to capitalize on any advantage, however small, that could increase their performance. This review focuses on several topics related to the question of whether the gut microbiome may be used to predict performance response to dietary and/or training interventions. This includes topics such as (1) the effect of exercise on the gut microbiome, (2) the effect of dietary components or patterns relevant to athletic nutrition on the gut microbiome, and (3) the effect of the gut microbiome on performance response to diet and exercise. Each of these related topics will be discussed, as will gaps in the research and future directions.

## Methods

Numerous reviews have been published highlighting the effect of exercise on the gut microbiota (39–56). However, the primary focus of these reviews has been the implications for aspects of host health, such as the immune system and risk of chronic diseases. Only a few have discussed the implications for athletic performance (41, 48, 52). This review aims to provide a more in-depth discussion of the interactive effect between the gut microbiota and diet on athletic performance and highlight the need for further research in this area. A literature search in PubMed and Google Scholar, including combinations of key words “gut microbiota”, “exercise”, “performance,” “variability,” and “effect,” was used to identify relevant studies. References were also obtained from the above review articles.

The earliest study found was published in 2008 by Matsumoto et al. (57) but was followed by a host of studies aiming to identify the effects of exercise on the gut microbiota (19, 58–83). The majority of these studies have investigated the effect of exercise on the gut microbiota in rodents (57–75), though some have studied humans in intervention trials (18, 76–78, 84) and in cross-sectional or observational comparisons of athletes or active individuals and sedentary individuals (19, 79–83, 85, 86).

## Results

### The Effect of Exercise on the Gut Microbiome

#### Microbiota Features Affected by Exercise

Table 1 summarizes the studies listed above and their findings of the effect of exercise on the gut microbiota.

**Table 1:** Summary of effect of exercise on the gut microbiome.

**References**	**Subjects**	**Type of exercise**	**Type of study**	**Diet**	**Microbiota method**	**Microbial diversity**	***Firmicutes* (phylum)**	***Bacteroidetes* (phylum)**	***Lactobacillaceae* (family)**	***Bifidobacteriaceae* (family)**	**Other microbiota factors**
**Rodent studies**											
Allen et al. (59)	Mice (C57BL/6J, 6 wk, male)	Voluntary wheel running (VWR) vs. forced treadmill running (FTR) for 6 wk	Intervention	Commercial diet	Composition (16S)	↓ in VWR (Chao1) ↔ (Shannon)	↓ *Turicibacter* in VWR				
Lambert et al. (62)	Mice (diabetic db/db C57BL/KsJ-leprdb/leprdb and normal db/+, 6 wk, male)	Treadmill	Intervention	Chow diet	Composition (qPCR)		↑ *Firmicutes, Clostridium*	↓ *Bacteroides, Prevotella*	↑ *Lactobacillus* (not after adjustment for body weight and blood glucose)	↑ *Bifidobacterium* in exercised normal vs. sedentary normal ↓ *Bifidobacterium* in exercised diabetic	↓ *Enterobacteriaceae* in exercised diabetic vs. sedentary diabetic
Lamoureux et al. (63)	Mice (C57BL/6, 6–10 wks, 11 male and 31 female)	Voluntary exercise (VE) vs. moderate forced exercise (treadmill) (FE) for 8 wk	Intervention	Normal diet	Composition (16S)	↔α-diversity (species richness) or β-diversity (weighted and unweighted UniFrac, Bray-Curtis)					Random forest predicted voluntary exercise with 97% accuracy using *Bacteroides, Lactobacillus, Rikenellaceae, Lachnospiraceae*; predicted forced exercise with 86% accuracy using *Bacteroides, Clostridiales*, and *Lactobacillales*
Liu et al. (75)	Mice (C57BL/6J, 4 wk, male; myocardial infarction (MI), sham, or no-surgery)	Treadmill for 4 wk	Intervention	None	Composition (16S)	↑ α-diversity (Shannon, PD_whole_tree)					↑ *Butyricimonas, Prevotella, Akkermansia* in exercise/non-surgery mice ↑ *Parasutterella* in control/non-surgery mice ↑ *Erysipelotrichaceae, Sphingobacteriales, Akkermansia* in exercise/sham mice ↑ *Corynebacterium, Staphylococcus, Enterobacteriaceae* in control/sham mice ↑ *Phenylobacterium* and *Roseateles* in exercise/MI mice
Brandt et al. (73)	Mice (C57BL/6N,8–10 wk, male, loxP insertions in Ppargc1a gene)	Voluntary wheel running (VWR) for 16 wk	Intervention	Standard rodent chow (CON) vs. High-fat diet (HFD) vs. HFD + resveratrol	Composition (16S)	↓ α-diversity in HFD mice vs. CON ↑ β-diversity in HFD mice vs. CON		↑ *Bacteroidetes* in HFD plus exercise vs. HFD		↓ *Actinobacteria* in HFD plus exercise vs. HFD	↓ *Erysipelotrichaceae, Verrumicrobioa* in HFD plus exercise vs. HFD ↑ *Alistipes* in HFD plus exercise vs. HFD
Campbell et al. (67)	Mice (C57BL/6NT, 8 wk, male)	Voluntary wheel running for 12 wk	Intervention	Normal diet vs. High-fat diet	Composition (TRFLP, 16S)		↑ *Allobaculum, Clostridiales, Faecalibacterium prausnitzii*				
Evans et al. (64)	Mice (C57BL/6J, 6 wk, male)	Voluntary wheel running for 12 wk	Intervention	Low-fat vs. High-fat diet	Composition (16S, qPCR, TRFLP)	↑ α-diversity (Shannon) with high-fat diet and exercise	↓ *Firmicutes* (16S), *Turicibacteraceae, Erysipelotrichaceae* (qPCR) ↔*Firmicutes* (qPCR) ↑ *Bacteroidetes*/ *Firmicutes* (qPCR) ↓ *Bacteroidetes*/ *Firmicutes* (qPCR)	↑ *Bacteroidetes* (16S), *Bacteroidetes*/ *Firmicutes* (qPCR) ↔*Bacteroidetes* (qPCR) ↓ *Bacteroidetes*/ *Firmicutes* (qPCR)	↓ *Lactobacillaceae* (qPCR)	↓ *Actinobacteria* (16S), *Bifidobacteriaceae* (qPCR)	↑ butyrate-producing taxa
McCabe et al. (74)	Mice (C57BL/6J, 6 wk, male)	Voluntary wheel running for 14 wk	Intervention	Low-fat vs. High-fat diet	Composition (16S)		↓ *Firmicutes*/ *Bacteroides* in HF-exercise	↓ *Firmicutes/* *Bacteroides* in HF-exercise			
Kang et al. (61)	Mice (C57BL/6J, 8 wk, male)	Motorized wheel running for 16 wk	Intervention	Normal diet vs. High-fat diet	Composition (16S)		↑ *Firmicutes, Lachnospiraceae* ↓ *Streptococcaceae*	↓ *Bacteroidetes*			↓ *Tenericutes*
Denou et al. (70)	Mice (C57BL/6J, 8 wk, male)	High-intensity interval training (HIIT) on treadmill for 6 wk	Intervention	Chow diet vs. High-fat diet	Composition (16S) and function (PICRUSt)	↑ α-diversity (Shannon)	↑ *Bacteroidetes*/ *Firmicutes*	↑ *Bacteroidetes*/ *Firmicutes, Bacteroidales*			↑ KEGG-annotated metabolism genes
Choi et al. (60)	Mice (C56BL/6NT, 11–13 mo, male)	Voluntary wheel running for 5 wk	Intervention	Polychlorinated biphenyls (PCBs)	Composition (16S)	↑ abundance	↑ *Firmicutes* (mostly *Lactobacillales*)		↑ *Lactobacillales*		↓ *Tenericutes* (*Erysipelotichaceae*) ↑ *Proteobacteria* (prevented PCB-induced decrease)
Liu et al. (65)	Rats (ovariectomized (OVX) high capacity (HCR) and low capacity (LCR) runners, 27 wk, females)	Voluntary wheel running for 11 wk	Intervention	Chow diet	Composition (16S)	↔α-diversity (Chao1)	↑ *Firmicutes* in HCR ↓ *Firmicutes* in LCR				↓ *Proteobacteria, Cyanobacteria* in HCR ↑ *Proteobacteria, Cyanobacteria* in LCR
Mika et al. (50)	Rats (F344, day 24 vs. day 70, male)	Voluntary wheel running for 6 wk	Intervention	Standard diet	Composition (16S)	↓ α-diversity (Shannon entropy, species richness) in young rats ↑ β-diversity (unweighted UniFrac) in young rats	↓ *Firmicutes* in young rats ↑ *Blautia, Anaerostipes* in young rats ↑ *Turicibacter* in adult rats	↑ *Bacteroidetes* in young rats			↑ *Euryarchaeota* (*Methanosphaera*) in young rats ↓ Proteobacteria (*Desulfovibrio*) in young rats ↓*Rikenellaceae* in young rats ↑ *Rikenellaceae* in adult rats *Bacteroides, Bifidobacterium, Ruminococcus, Rikenellaceae, Parabacteroides, Christensenellaceae, Methanosphaera* predict time point in young rats
Matsumoto et al. (57)	Rats (Wistar, 7 wk, male)	Voluntary wheel running for 5 wk	Intervention	Casein-sucrose diet	Composition (PCR-TGGE)						Differential clustering between exercise and controls ↑ butyrate-producing taxa
Queipo-Ortuno et al. (58)	Rats (Sprague Dawley, 5 wk, male)	Voluntary wheel running for 6 d	Intervention	Activity based anorexia (ABA, 1 h food intake w/ exercise), ABA control (sedendary), Exercise (ad lib w/ exercise), Ad lib (ad lib sedentary)	Composition (PCR-DGGE, qPCR)	↓ α-diversity (band richness)	↓ *Firmicutes* in ABA vs. Exercise and Ad lib; in Exercise vs. Ad lib ↑ *Clostridium* in ABA ↑ *B. Coccoides-E. rectale* group in Exercise vs. Ad lib ↓ *Clostridium, Enterococcus* in Exercise vs. Ad lib	↓ *Bacteroidetes* in ABA vs. Exercise and Ad lib ↑ *Bacteroides, Prevotella* in ABA vs. ABA control ↓ *Bacteroides, Prevotella* in Exercise vs. Ad lib	↑ *Lactobacillus* in Exercise vs. Ad lib	↓ *Actinobacteria* in ABA ↑ *Actinobacteria* in Exercise vs. Ad lib ↑ *Bifidobacterium* in Exercise	↑ *Proteobacteria* in ABA vs. Exercise and Ad lib
Welly et al. (69)	Rats (obesity prone OP-CD, 4 wk, male)	Voluntary wheel running	Intervention	High-fat diet (HFD; groups: sedentary, w/ exercise, weight matched to exercise)	Composition (qPCR)	↔α-diversity (species richness)	↑ *Streptococcaceae* in Exercise ↔*Firmicutes*/ *Bacteroidetes* ratio	↓ S24–7 in Exercise ↓ *Bacteroidetes* in Exercise and weight-matched (trending, not significant) ↔*Firmicutes*/ *Bacteroidetes* ratio			↓ *Rikenellaceae* in Exercise
Feng et al. (71)	Rats (high capacity (HCR) and low capacity (LCR) runners, sugery or sham)	Treadmill for 6 wk	Intervention	None	Composition (16S)	↑ α-diversity (Shannon) in LCR rats ↑ β-diversity in LCR and HCR rats	↑ Firmicutes in HCR rats	↓ Bacteroidetes in HCR rats			
Petriz et al. (68)	Rats (Zucker (obese), Zucker (spotaneously hypertensive), and Wistar (non-obese, control), 20 wk, male/female?)	Forced treadmill running for 4 wk	Intervention	Not reported	Composition (16S)	↑ α-diversity (Shannon, rarefaction)	↑ *Firmicutes* ↓ *Streptococcus* in non-obese Wistar ↑ *Allobaculum* in hypertensive	↓ *Bacteroidetes* in non-obese Wistar	↑ *Lactobacillus* in obese Zucker		↓ *Proteobacteria* ↓ *Sutterella, Aggregatibacter* in hypertensive
Batacan et al. (72)	Rats (Wistar, 12 wk, male)	Control (CTL), sedentary (SED), light-intensity trained (LIT), and high-intensity interval trained (HIIT) for 12 wk	Intervention	Standard chow (SC) versis high-fat high-fructose (HF) diet	Composition (16S)	↔α-diversity between activity groups regardless of diet (Chao1, observed species, Shannon, Simpson,	↓ *Firmicutes* in LIT-SC ↑ *Clostridiaceae* in HF ↑ *Lachnospiraceae* in HIIT-SC		↑ *Lactobacillus johnsonii* increased in LIT-SC	↑ *Actinobacteria* in LIT-SC ↓ *Bifidobacterium* in HF	↑ *Tenericutes, Prevotella excrementihominis, Erysipelotrichaceae* in LIT-SC (*Erysipelotrichaceae* higher in HF-fed rats) ↓ *Turicibacteraceae* in SC ↑ polysaccharide degraders and SCFA producers with exercise in both SC and HF (effects weaker in HF)
						dominance, richness, equitability, evenness)↑β-diversity (weighted and/or unweighted UniFrac)					
**Human intervention studies**											
Allen et al. (18)	Humans (32 previously sedentary subjects, 18 lean 14 obese)	Endurance exercise for 6 wk progressed from moderate to vigorous; followed by 6 wk sedentary	Intervention	Habitual diet	Composition (16S) and function (qPCR of select functional genes)	↔α-diversity (Chao1) ↔β-diversity after exercise/ washout (weighted and unweighted UniFrac) β-diversity different between lean/obese at baseline					↑ butyrate-regulating group in lean and obese Butyrate group explained 61.2% of variance in microbiota response and 84% of VO_2max_ response ΔButyrate-producers ~Δlean mass
Munukka et al. (76)	Humans (19 overweight, sedentary women)	Endurance exercise (bike erg) for 6 wk	Intervention	None	Composition and function (16S, metagenomics)	↔α-diversity (not reported) ↑ β-diversity (Jaccard)	↔*Firmicutes*	↔*Bacteroidetes*		↑ *Bifidobacteriaceae* (dependent on weight, body fat %, android fat %, total energy intake, sucrose, fiber)	↓ *Proteobacteria* ↑ *Verrumicrobiaceae, Akkermansia* ↓ genes in fructose, mannose, alanine, aromatic amino acid metabolism
Taniguchi et al. (77)	Humans (31 Japanese adult males, >60 years old)	Cycling for 5 wk, no washout between intervention and 5 wk control period	Intervention	Habitual diet	Composition and function (16S, metagenomics)	↔α-diversity (Shannon, observed OTUs)	↓ *Clostridium* difficile during exercise				↑ *Oscillospira* during exercise (no longer significant after adjusting for dietary changes and treatment sequence) ↑ Metagenomic functions belonging to “Genetic Information Processing” and “Nucleotide Metabolism” during exercise
Morita et al. (84)	Humans (32 Japanese sedentary adult women, >65 years old)	Aerobic exercise (AE) or trunk muscle training (TM) for 12 wk	Intervention	Habitual diet	Composition (TRFLP)		↓ *Clostridium* subcluster XIVa decreased in AE ↑ *Clostridium* IX in TM	↑ *Bacteroides* (negatively correlated with pre-*Bacteroides*)			
Cronin et al. (78)	Humans (74 healthy Irish adults)	Mixed aerobic and resistance exercise training program for 8 wk	Intervention	Whey Protein+ Exercise (EP) vs. Exercise (E) vs. Whey Protein (P)	Composition and function (metagenomics)	↔α-diversity from baseline but higher in EP vs. P group					Differential abundance of virus species between groups
						after intervention ↓ Archaea diversity in E group alteration in β-diversity of the gut virome in P and EP groups					
**Human cross-sectional studies**											
Bressa et al. (82)	Humans [40 premenopausal Caucasian women; 19 active (ACT), 21 sedentary (SED)]	General physical activity (measured for 1 wk)	Cross-sectional	Habitual diet	Composition (16S, qPCR)	↔α-diversity (Chao1, Observed, Shannon) ↔β-diversity (unweighted or weighted UniFrac) Observed # species, Shannon, Simpson indices (+) ~ minimum time per sedentary bout (work days)	↑ *Firmicutes* (trending) ↔ F/B ratio	↓ *Bacteroidetes* (trending) ↔ F/B ratio		↑ *Bifidobacterium* in ACT women	↑ *Haemophilus, Paraprevotella, Coprococcus, Ruminococaceae unclassified 1* in ACT women (16S) ↓ *Desulfovibrionaceae unclassified, Turicibacter, Barnesiellaceae, Odoribacteriaceae, Ruminococcaceae unclassified 2, Ruminococcus* in ACT women (16S) ↑ *Faecalibacterium prausnitzii, Roseburia hominis, Akkermansia mucinipihila* in ACT women (qPCR)
Karl et al. (86)	Humans (73 Norwegian soldiers, 26 provided pre- and post- stool samples)	4-day cross country ski-march (STRESS)	Cross-setional	Rations with or without protein- or carbohydrate-based supplements	Composition (16S)	↑ α-diversity post-STRESS (Shannon)	↑ *Firmicutes*	↓ *Bacteroidetes*			Random forest using microbiota predicted pre- and post-STRESS samples with 100% accuracy
Shukla et al. (81)	Humans (10 myalgic encephalomyelitis/ chronic fatigue syndrome (ME/CFS) patients, 10 healthy controls)	Cycling (max test)	Cross-sectional	Habitual diet	Composition (16S) in blood and stool		↑ *Firmicutes* in ME/CFS patients after exercise ↓ *Firmicutes* in healthy controls after exercise ↑ *Firmicutes*/ *Bacilli* and *Clostridium* in blood of ME/CFS patients after exercise (not in healthy controls)	↓ *Bacteroidetes* in ME/CFS patients after exercise ↑ *Bacteroidetes* in healthy controls after exercise			
Barton et al. (79)	Humans (40 professional rugby players, 46 controls)	Rugby	Cross-sectional	None	Function (metagenomics)	↑ α-diversity in athletes vs. high-BMI controls (Shannon, Simpson) or all controls (Phylogenetic diversity, Chao1, Observed species)					↑ *Akkermansia* pathways in athletes vs. high-BMI controls ↑ pathways (amino acid and antibiotic biosynthesis, carbohydrate metabolism) in athletes
Clarke et al. (19)	Humans (40 professional rugby players, 46 controls)	Rugby	Cross-sectional	Habitual diet	Composition (16S)	↑ α-diversity in athletes vs. high-BMI controls (Shannon, Simpson) or all controls (Phylogenetic diversity, Chao1, Observed species)	↑ *Firmicutes* in athletes vs. high-BMI controls	↓ *Bacteroidetes* in athletes vs. high-BMI controls			↑ *Akkermansia* in low-BMI athletes vs. high-BMI controls
O'Donovan et al. (83)	Humans (37 professional Irish athletes)	16 different sports across varying sports classification groups (SCGs)	Cross-sectional	Habitual diet	Composition and function (metagenomics)	↔α-diversity (Shannon and Simpson) between SCGs		↑ *Bacteroides caccae* ~ SCGs with high static/high dynamic components		↑ *Bifidobacterium animalis* ~ SCGs with low static/high dynamic components	↑ *Streptococcus suis, Clostridium bolteae, Lactobacillus* phage *LfeInf, Anaerostipes hadrus*, flavin biosynthesis and fermentation pathways ~ SCGs with moderate dynamic component ↑ *Lactobacillus acidophilus, Prevotella intermedia, Faecalibacterium prausnitzii* ~ SCGs with low static/high dynamic components ↑ folate and amino acid biosynthesis pathways ~ SCGs with high static/high dynamic components ↑ Nucleotide biosynthesis ~ SCGs with high static/low dynamic components No species associated with SCGs with high static/low dynamic components or high dynamic/moderate static components
Petersen et al. (80)	Humans (33 amateur and professional cyclists)	Cycling	Cross-sectional	None	Composition and function (16S, metagenomics, RNA-Seq)	↑ α-diversity (Shannon, # of genera) in Cluster 3 (contains more professional cyclists vs. amateur)		↑ *Prevotella* with increased exercise load (hrs per week) ↓ *Bacteroides* in athletes (though no non-athlete control for comparison)			↑ *Methanobrevibacter smithii* gene expression in professional cyclists

*ABA, activity-based anorexia; ACT, active [designated group in Bressa et al. (82)]; AE, aerobic exercise training; BMI, body mass index; FE, forced exercise; FTR, forced treadmill running; HCR, high-capacity running; HFD, high-fat diet; HIIT, High intensity interval training; KEGG, Kyoto Encyclopedia of Genes and Genomes; LCR, low-capacity running; OTU, operational taxonomic unit; OVX, ovariectomized; PCB, polychlorinated biphenyls; PCR-DGGE, polymerase chain reaction denaturing gradient gel electrophoresis; PCR-TGGE, polymerase chain reaction temperature gradient gel electrophoresis; PICRUSt, Phylogenetic Investigation of Communities by Reconstruction of Unobserved States; qPCR, quantitative polymerase chain reaction; SCG, sports classification group; SED, sedentary [designated group in Bressa et al. (82)]; TM, truck muscle training; TRFLP, Terminal restriction fragment length polymorphism; VE, voluntary exercise; VO_2max_, maximal (O_2_) oxygen uptake; VWR, voluntary wheel running*.

Although there are similarities in microbial factors shown to be affected by exercise within the literature, directions of the effects are inconsistent, and some studies show contradictory results. For example, while some studies show a reduction in *Firmicutes* and/or an increase in *Bacteroidetes* as a result of exercise (58, 64, 66, 70, 73, 74, 81, 84), others show the opposite effect (19, 60–62, 68, 71, 81, 86), and others show no effect (69, 76, 82).

Findings on the effect of exercise on measures of diversity are also highly variable, some showing increases in α-diversity (19, 60, 64, 68, 70, 71, 75, 79, 86), some showing decreases (58, 59, 66), and others reporting no difference (18, 59, 63, 65, 69, 76–78, 82, 83). Brandt et al. (73) also found that exercise attenuated the decrease in α-diversity that occurred when mice were fed a high-fat diet.

Bacterial taxa commonly shown to respond to exercise training include *Lactobacillus* (typically increased) (58, 60, 62, 68)*, Bifidobacterium* (typically increased) (58, 62, 76, 82), *Proteobacteria* (typically decreased) (58, 65, 66, 68, 76), *Akkermansia* (typically increased) (19, 75, 76, 79, 82), *Streptococcus* (variable effects) (61, 68, 69), *Clostridium* (variable effects) (58, 62, 63, 67, 77), *Turicibacter* (typically decreased) (59, 64), and *Rikenellaceae* (typically decreased) (63, 66, 69) as well as measures of α- and β-diversity (variable effects) (19, 58, 60, 64, 66, 68, 70, 76, 80).

However, in some studies, changes in taxa are dependent on other factors such as changes in weight, body fat, and blood glucose (64, 76). This suggests, that the associated metabolic effects of the exercise regime may be the proximal cause, while exercise is the ultimate cause. Related variables, SCFA production and butyrate-producing taxa, have been consistently shown to increase in response to exercise (18, 57, 79) and have also been positively correlated to changes in lean muscle mass, also suggesting that SCFAs may play an important role in mediating the effects of exercise and the gut microbiome on host response (18). Table 2 summarizes additional effects of exercise on microbial metabolites, host health, and dietary interactions.

**Table 2:** Summary of effect of exercise on the microbial metabolites, host health, and dietary interactions.

**References**	**SCFAs**	**Other metabolites**	**Host health**	**GI tract physiology**	**Diet interactions**
**Rodent studies**					
Allen et al. (59)				↓ gastrointestinal inflammation in VWR, ↑ in FTR	
Lambert et al. (62)			↑ glucose in exercised normal vs. sedentary normal ↔ glucose in exercised diabetic vs. sedentary diabetic		
Liu et al. (75)			↑ Left ventricular ejection fraction (EF), fractional shortening (FS), cardiac output (CO), and stroke volume (SV) FS, EF, CO, SV, and left ventricular end systolic diameter (LVESD) correlated with gut microbiota taxa		
Brandt et al. (73)			↑ body weight in HFD vs. CON, HFD plus resveratrol ↔ body weight in HFD vs. HFD plus exercise ↑ body fat %, subcutaneous, and visceral adipose tissue in HFD and HFD plus exercise vs. CON ↓ lean body mass in HFD and HFD plus exercise vs. CON ↑ serum amyloid A (SAA) in all HFD groups vs. CON		
Campbell et al. (67)			Body fat %: High-fat sedentary > High-fat exercise > low-fat sedentary > low-fat exercise	↓ inflammatory infiltrate, COX-2, and high-fat diet-induced morphological changes in exercise	
McCabe et al. (74)			↑ Bone volume fraction with exercise [~ Firmicutes/Bacteroides (–), *Clostridia* (–), *Lachnospiraceae* (–), *Actinobacteria* (+)] ↓ Trabecular bond volume with high-fat diet ↑ Marrow adiposity with high-fat diet ↓ Body weight, fat pad mass, fasting glucose with exercise and low-fat diet		
Kang et al. (61)			↔ high-fat diet-induced anxiety ↑ cognitive abilities		
Denou et al. (70)			High-fat diet with exercise vs. high-fat diet: ↔ body mass, fasting blood glucose ↑ insulin tolerance, RER, food intake, time to exhaustion with exercise		
Liu et al. (65)		↓ non-esterified fatty acids (NEFAs) and triglycerides in LCR rats ↑ non-esterified fatty acids (NEFAs) and triglycerides in HCR rats	↓ body weight, fat mass, feed efficiency in LCR rats ↑ body weight, fat mass, food intake, feed efficiency of HCR rats		*Christensenellaceae* ~ food intake
Mika et al. (50)			↓ weight in adult rats ↑ weight, lean mass in young rats		
Matsumoto et al. (57)	↑ cecal n-butyrate			↑ cecum size/weight	
Queipo-Ortuno et al. (58)			↑ body weight in Exercise and Ad lib ↓ body weight in ABA and ABA control ↑ ghrelin [~*Lactobacillus* (–), *Bifidobacterium* (–)]		
			↓ leptin [~*Lactobacillus* (+), *Bifidobacterium* (+)]		
Welly et al. (69)			↓ total cholesterol, adiposity, inflammation in Exercise and weight-matched sedentary ↑ total energy expenditure in Exercise and weight-matched sedentary ↓ RQ, insulin resistance, LDL, liver mass in Exercise ↑ mitochondrial function in brown adipose in Exercise		
Feng et al. (71)			Exercise improved preoperative cognitive impairment in LCR rats		
Petriz et al. (68)			↑ velocity ↓ lactate [~*Clostridiaceae* (–), *Bacteroidaceae* (–), *Osillospira* (+), *Ruminococcus* (–)]		
Batacan et al. (72)					Samples grouped by diet (not activity group)
**Human intervention studies**					
Allen et al. (18)	↑ SCFAs in lean ΔButyrate ~Δlean mass		↑ lean body mass, bone mineral density, VO_2max_ ↓ body fat % ΔButyrate and butyrate-producers ~Δlean mass		
Munukka et al. (76)			↑ max power, VO_2max_, glucose ↓ lactate, HDL, LDL, large VLDL ↔ weight, blood pressure, weight circumfrence, BMI, fat mass/%		
Taniguchi et al. (77)			↑ VO_2peak_ [~C. difficile (–)], HDL [~*Oscillospira* (+)], total cholesterol [~*C. difficile* (–)] during exercise ↓ CAVI [~C. difficile (+)], intrahepatic fat %, HbA1c [~*Oscillospira* (–), ~*C. difficile* (+)] during exercise ↔ body fat % [~*Oscillospira* (–)]; visceral fat area, SBP, AST, ALT [~*C. difficile* (+)]; LDL [~*C. difficile* (–)]		*Oscillospira* ~ changes in light-colored vegetable, seaweed, and rice consumption
Cronin et al. (78)			↑ VO2max, lean mass in E and EP groups ↓ resting heart rate, % body fat, total fat mass, truck fat mass in E and EP groups ↔ pro-inflammatory markers		
**Human cross-sectional studies**					
Bressa et al. (82)		↑ cysteine aminopeptidase in ACT women [~*Bacteroides* (–)] ↔α-fucosidase [~*Bifidobacterium* (+), *Odoribacter* (+)], alkaline phosphatase [~*Desulfovibrio* (–)]	↔ BMI, weight, adiposity and muscle parameters Turicibacter (–) ~ BMI *Barnesiellaceae* (+) ~ % body fat Odoribacter (+), Haemophilus (–) ~ adiposity index, estimated visceral fat, % body fat *Faecalibacterium* (+) ~ muscle mass index, appendicular muscle mass index *Coprococcus* (+), *Lachnospiraceae* unclassified 1 (+) ~ appendicular muscle mass index		*Turicibacter* ~ dairy products, cereals *Bifidobacterium* ~ protein intake % *Odoribacter* ~ fiber, fat intake % *Ruminococcaceae* unclassified 1&2 ~ fat intake %
Karl et al. (86)		Random forest using stool metabolites predicted pre- and post-STRESS samples with 84% accuracy		↑ intestinal permeability (IP) Pre-STRESS *Actinobacteria*	
		81% of stool metabolites decreased during STRESS 478 plasma metabolites significantly changed during STRESS, including metabolites partially or fully derived from microbial metabolism		and changes in serum IL-6 and stool cysteine accounted for 84% of variability in change in IP	
Barton et al. (79)	↑ SCFAs in athletes	In athletes: ↑ TMA, TMAO, L-carnitine, dimethylglycine, O-acetyl carnitine, proline betaine, creatine, acetoacetate, 3-hydroxy-isovaleric acid, acetone, N-methylnicotinate, N-methylnicotinamide, phenylacetylglutamine (PAG), 3-methylhistidine, lysine, and methylamine ↓ glycerate, allantoin, succinate, glycine, tyrosine			Propionate ~ protein Butyrate ~ dietary fiber
Clarke et al. (19)					Diversity ~ protein intake and creatine kinase
O'Donovan et al. (83)		21 metabolites significantly different between SCGs (4 with significant pairwise differences: succinic acid, cis-aconitate, lactate, and creatinine) ↑ cis-aconitate, succinic acid in SCG with moderate static/high dynamic vs. low static/high dynamic ↑ fecal creatinine in SCG with low static/high dynamic vs. high static/low dynamic and moderate/high static and high dynamic ↓ lactate in SCG with low static/high dynamic vs. moderate/high static and high dynamic			

*ABA, activity-based anorexia; ACT, active [designated group in Bressa et al. (82)]; ALT, alanine aminotransferase; AST, aspartate aminotransferase; BMI, body mass index; CAVI, cardio-ankle vascular index; CO, cardiac output; CON, control; E, Exercise [designated group in Cronin et al. (78)]; EF, left ventricular ejection fraction; EP, Whey protein + Exercise [designated group in Cronin et al. (78)]; FS, fractional shortening; FTR, forced treadmill running; HCR, high-capacity running; HDL, high-density lipoprotein; HFD, high-fat diet; LCR, low-capacity running; IP, intestinal permeability; LDL, low-density lipoprotein; LVESD, left ventricular end systolic diameter; NEFA, non-esterified fatty acid; PAG, phenylacetylglutamine; RER, respiratory exchange ratio; RQ, respiratory quotient; SAA, serum amyloid A; SBP, systolic blood pressure; SCFA, short-chain fatty acid; SV, stroke volume; TMA, trimethylamine; TMAO, trimethylamine-N-oxide; TRFLP, VLDL, very low-density lipoprotein; VO_2max_, maximal (O_2_) oxygen uptake; VO_2peak_, highest value of VO_2_ achieved during high-intensity exercise test*.

#### Potential Causes of Discrepancies Between Studies

**Figure 2:**
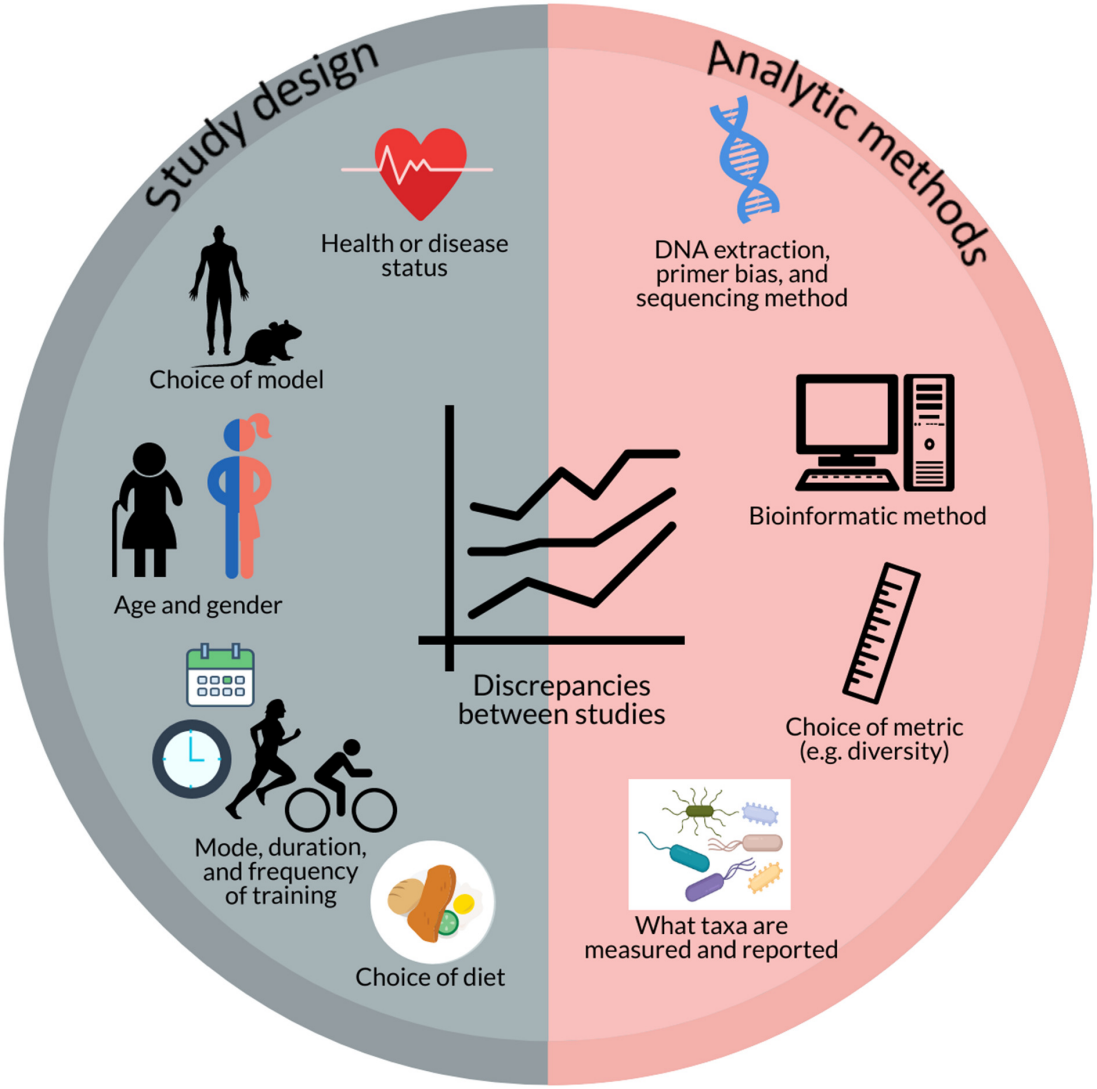
Potential factors contributing to discrepancies between studies investigating the effect of exercise on the gut microbiome include aspects of study design (e.g., health or disease status; choice of model; age and gender; mode, duration, and frequency of training; and choice of diet) and analytic methods (e.g., DNA extraction, primer bias, and sequencing method; bioinformatic method; choice of metrics; and what taxa are measured and reported).

Potential reasons for the disparate results of these studies include study design factors as well as analytic methods (Figure 2). Study design factors include the choice of model (e.g., humans, mice, rats), the strain of mouse/rat (e.g., C57BL/6J, Zucker, Wistar, Sprague Dawley, etc.) (87), choice of diet (72), health or disease status (75, 81), age (66), gender, and the mode, duration, and intensity of training (59, 63, 72, 83, 84) as well as analytic methods such as DNA extraction and PCR primer biases (88–90), choice of microbiome sequencing methods (e.g., 16S rRNA gene sequencing, qPCR, metagenomics, etc.) (64), bioinformatic pipelines (90), and choice of diversity metrics (e.g., Shannon, Chao1, Simpson, etc.). For example, mouse models often use forced treadmill running or voluntary wheel running as modes of training. However, forced treadmill exercise often uses aversive motivation, such as shocks, which could induce negative stress responses (91–93) that may also affect intestinal permeability and the gut microbiome (59, 94, 95). In humans, exercise or sport is a broad term that can apply to a wide range of modes, durations, and intensities of activity. O'Donovan et al. (83) attempted to determine differential effects of different modes of exercise on the gut metagenome by doing a cross-sectional analysis of professional athletes from different sports with varying degrees of static and dynamic components. In this analysis, O'Donovan etal. found some differences in bacterial taxa and metabolites between sports classification groups (SCGs) that did not correlate with any other metadata (e.g., diet, sex, etc.) (83).

In addition to differences in *how* results are obtained or measured, there is also a great deal of heterogeneity in *what* results are measured, or reported, that make it difficult to determine the full extent of variability in response between studies. In order to gain better insight into the potential effects and pathways by which exercise exerts its effect on the gut microbiome, it would be beneficial for studies to report effects on *at least* a certain standard set of microbiota variables that have already been shown to be relevant by multiple studies such as *Firmicutes, Bacteroidetes, Lactobacillus, Bifidobacterium, Akkermansia, Clostridium*, and *Proteobacteria* as well as diversity (though a standard metric has yet to be determined), butyrate-producing taxa (96), and SCFA production, even if the result is no change/difference. Munukka et al. (76) reported a lack of consistent effects due to inter-individual variability in response of the gut microbiota to exercise. This too is an important finding that should be reported and explored to determine factors that contribute to this variability in response and whether these differences in microbial response translate to differences in physiological response. These reporting standards would allow for better comparison between studies and potentially enable researchers to determine how different methods impact the results and elucidate factors that may contribute to variability in response.

### Effect of Dietary Components Relevant to Exercise Nutrition on the Gut Microbiome

#### Confounding Effects of Diet

**Figure 3:**
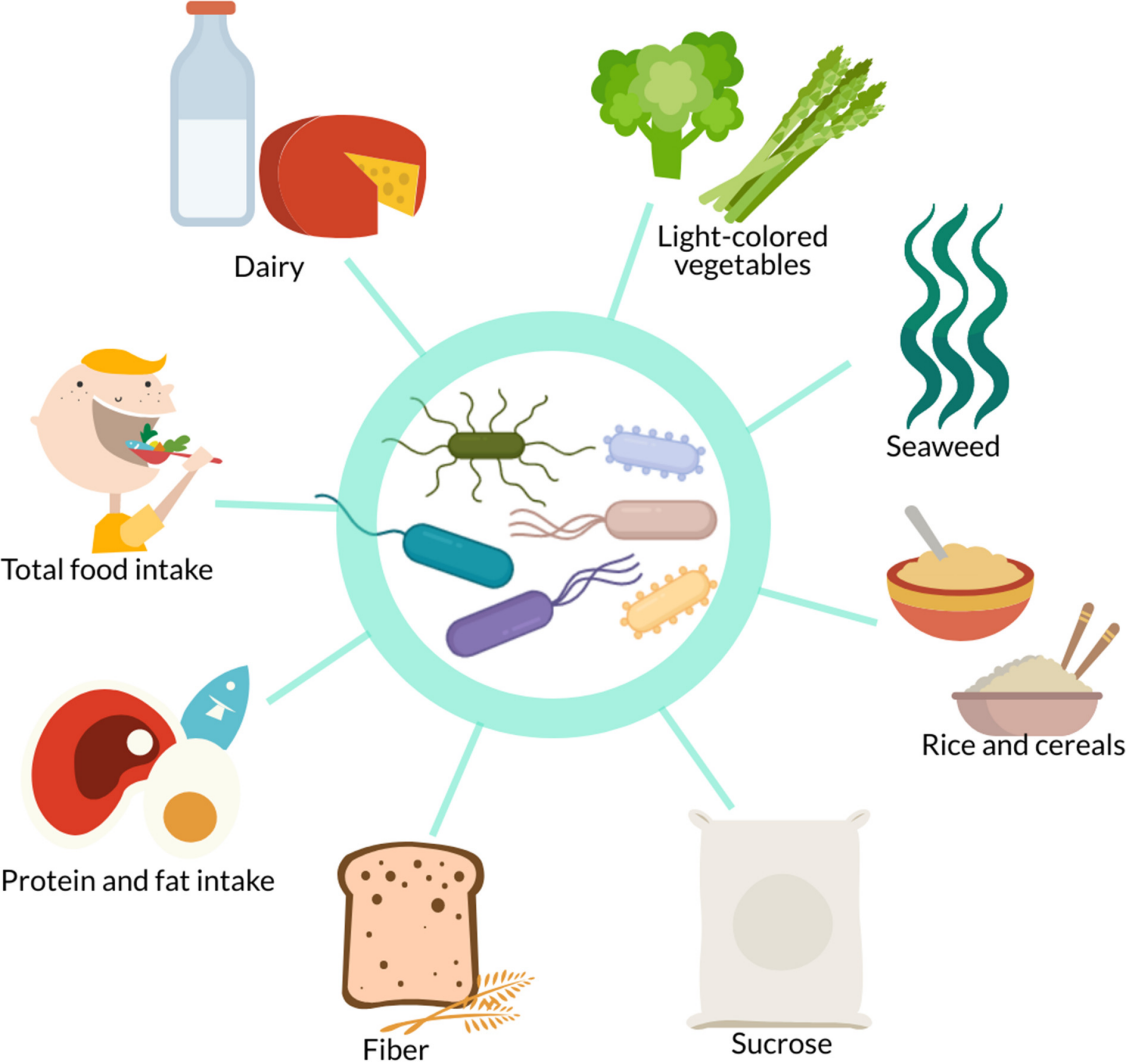
In studies investigating the effect of exercise on the gut microbiome, confounding dietary factors include dairy, light-colored vegetables, seaweed, rice, cereals, sucrose, fiber, protein intake, fat intake, and total food intake.

Diet is also a major factor that influences and shapes the gut microbiome (15–17). Kang et al. report that diet and exercise both cause shifts in the gut microbiome but that these changes are orthogonal (61). However, some of the studies above reported that dietary factors influenced the gut microbiota independently of, or in combination with, exercise. Dietary factors found in the studies presented here to associate with gut microbiome differences or changes include dairy products (82), light-colored vegetables (77), seaweed (77), rice (77), cereals (82), sucrose (76), fiber (76, 79, 82), protein intake (19, 79, 82), fat intake (82), and total food intake (65, 76) (Figure 3). Some differences or changes in the gut microbiota that seem to be associated with exercise may therefore be due to differences or changes in dietary intake, especially plants and carbohydrates, rather than exercise itself. There is therefore a need for studies investigating the link between the gut microbiome and exercise that control and standardize the dietary intake of participants.

#### Effects of Supplements and Dietary Patterns on the Gut Microbiome

Although studies have shown some dietary interactions with the gut microbiome in athletes, it is unclear the extent to which the gut microbiome might be affected by supplements or dietary patterns commonly used by athletes, and the potential effects of this on the host. A review by Kårlund et al. (97) comprehensively discusses the topic of protein supplementation in athletes and the potential unknown effects on the gut microbiome. Excess protein may be fermented in the large intestine by various species from the genera *Clostridium, Bacteroides*, and others from the *Proteobacteria* phylum (98, 99), resulting in end products such as ammonia, amines, phenols, and sulfides as well as some SCFAs that may have systemic and metabolic effects on the host (100, 101). Different protein types have been shown to have differential effects on the gut microbiome (102–104) and plant-based vs. animal-based diets have also been shown to induce differences in the gut microbiome composition in humans (16). Additionally, different protein types have been assessed in the context of the anabolic response to exercise (105). However, there are no studies evaluating the impact of different types of protein supplements or whole-food protein sources on the gut microbiome and amino acid fermentation in athletes (97). As protein and protein supplements are widely advertised and recommended to athletes, this is an important gap in the research that should be addressed. Future research should also be sure to compare the effects of different protein sources as both isolated supplements as well as in their whole-food form as the matrix of the whole food has been shown to play an important role in the anabolic response to exercise and may alter effects based on factors such as the type and amount of fat (106, 107). Additionally, it would be interesting to know whether supplementation of plant-based proteins with amino acids such as leucine, lysine, and methionine, which is a strategy that has been shown by a couple studies to augment the anabolic effect of plant proteins (105), alters the effect of these proteins on the gut microbiome.

Carbohydrate is a primary fuel source for exercise and is therefore also a primary focus of athletes' dietary intake (108, 109). In addition to whole food forms of carbohydrates, such as bread, pasta, fruit, and potatoes, there is also a wide array of carbohydrate supplements that may be used before, during, or after exercise to enhance performance and recovery (110). The effects of whole food carbohydrates on the gut microbiome differs widely as a function of fiber content and type (111–113), though generally fiber tends to increase SCFA producing bacteria such as Bacteroidetes and Actinobacteria and decrease Firmicutes (114). However, the effects of frequent use of carbohydrate supplements, which are typically high in sugar and low in microbiota-accessible carbohydrates, on the gut microbiome is unknown. It is may therefore be of interest to develop carbohydrate supplements that also target the gut microbiome.

Though carbohydrates and protein are made the primary focus of athlete nutrition, fat is also an important fuel source during prolonged exercise and the popularity of high-fat diets such as the ketogenic diet has prompted athletes and scientists to investigate its potential for sports performance (115). However, evidence suggests that a high-fat diet does not improve exercise performance more than or as much as a high-carbohydrate diet (116). Additionally, the lack of microbiota-accessible carbohydrates on the ketogenic diet makes it questionable whether or how it would benefit the gut microbiome, though there has been little research in this area and none of it has been in athletes (117, 118).

Caffeine is also a widely used ergogenic aid among athletes. Coffee, one of the primary dietary sources of caffeine, has been linked to increases in *Bifidobacterium* and protection against high-fat diet-induced decreases in *Lactobacillus*, though these effects may be due to other bioactive compounds present in coffee such as chlorogenic acid (119). The effect of these aspects of dietary intake on the gut microbiome in athletes is only one half of the story. The other is the effect of the gut microbiome on the overall response, in terms of performance and training adaptation, of the athlete.

### Personalized Sports Nutrition and the Potential Effect of the Gut Microbiome on Response to Diet and Exercise

#### The Gut Microbiome in Personalized Sports Nutrition

As discussed in Hughes et al. (22, 23), the gut microbiome is a potential predictor of response to diet. However, that review focused on predictors of response relevant to general health and prevention of chronic disease. Here, the evidence that the gut microbiome may be a predictor of athletic performance is reviewed. Personalized sports nutrition has incorporated the type of sport or activity, the training status of the individual, the athlete's goals, the time of the competitive season, and the athlete's food preferences (120, 121) as well as biological traits such as genetic polymorphisms, RNA expression, and epigenetic modifications (28–30, 34–38) in the attempt to optimize athletic performance and response to training programs.

The gut microbiome should be incorporated into this system as it modulates metabolism of diet and dietary supplements, and therefore has the potential to contribute to variability in response. Inter-individual variability among athletes in response to dietary supplements, such as caffeine and antioxidants, has been attributed to genetic polymorphisms and baseline antioxidant concentrations (35, 122). However, the gut microbiome has been identified as an important factor in the bioavailability and metabolism of antioxidants (123–125) and may be involved in caffeine metabolism via mechanisms such as modulation of the expression of the N-acetyltransferase 2 (NAT2) gene (126). Variability in the gut microbiome has been linked to variability in serum carotenoid concentrations (127), which suggests that the gut microbiome does indeed play a role in modulating antioxidant metabolism. In short, the gut microbiome may affect the metabolism of dietary components, supplements, and dietary patterns marketed to and used by athletes, but this is an area of research that has not yet been adequately explored.

#### The Effect of the Gut Microbiome on Performance

Though the gut microbiome has been shown to modulate metabolism of relevant dietary components, as discussed above, the implications of this for performance are still unclear.

Mostly cross-sectional, studies have examined the correlation between measures of fitness, such as VO_2max_ and VO_2peak_, and the gut microbiota (18, 77, 85, 128–130) (Table 3). Butyrate-producing bacteria have been shown in both Allen et al. (18) and Estaki et al. (129) to correlate positively with VO_2max_ and VO_2peak_, respectively. *Bacteroides* and the *Firmicutes*/*Bacteroidetes* ratio have also been shown to correlate with VO_2max_ (85, 128, 130), although studies have shown contrasting results. Durk et al. (128) found the *Firmicutes*/*Bacteroidetes* ratio to correlate positively with VO_2max_. Conversely, Yu et al. (85) found a lower F/B ratio in elderly adults with higher exercise capacity and Yang et al. (130) found that the high VO_2max_ group had lower *Eubacterium rectale-Clostridium coccoides* (Erec), which are members of the *Firmicutes* phylum, and higher *Bacteroides*. Yu et al. (85) also identified several other taxa that were correlated with VO_2peak_ in their elderly population such as *Lactobacillales, Blautia, Ruminococcus, E. coli*, and *Alcaligenaceae*. Taniguchi et al. (77) found an inverse correlation between *Clostridium difficile* and changes in VO_2peak_ in elderly Japanese men during a cycling intervention.

**Table 3:** Summary of studies investigating the correlation between gut microbiota composition and measures of fitness.

**References**	**Subjects**	**Type of exercise**	**Type of study**	**Microbiota method**	**Performance metric(s)**	**Results**
Allen et al. (18)	Humans (32 previously sedentary subjects, 18 lean 14 obese)	Endurance for 6 wk progressed from moderate to vigorous; followed by 6 wk sedentary	Intervention	Composition (16S) and function (qPCR of select functional genes)	VO_2max_	Butyrate-regulating bacteria group explained 61.2% of variance in microbiota response and 84% of VO_2max_ response
Durk et al. (128)	Humans (healthy young adults)	Treadmill test	Cross-sectional	Composition (qPCR)	VO_2max_	*Firmicutes*/*Bacteroidetes* (F/B) ratio positively correlated to VO_2max_ VO_2max_ explained ~22% of variance in F/B ratio
Estaki et al. (129)	Humans (varying cardiorespiratory fitness levels)	Cycle ergometer	Cross-sectional	Composition (16S) and function (PICRUSt)	VO_2peak_	VO_2peak_ accounted for ~20% of variation in α-diversity and positively correlated with abundance of butryate-producing taxa VO_2peak_ + sex, fiber, and sugar intake explained 15.5% of variation in functional categories Protein associated with *Bacteroides* and explained 12.7% of taxonomic composition
Taniguchi et al. (77)	Humans (31 Japanese adult males, >60 years old)	Cycling for 5 wk, no washout between intervention and 5 wk control period	Intervention	Composition and function (16S, metagenomics)	VO_2peak_	Abundance of *Clostridium difficile* was negatively correlated with the increase in VO_2peak_ achieved during the exercise intervention
Morita et al. (84)	Humans (32 Japanese sedentary adult women, >65 years old)	Aerobic exercise (AE) or trunk muscle training (TM)	Intervention	Composition (TRFLP)	Trunk muscle strength (Kraus-Weber test), 6-min walk test (6MWT)	Abundance of *Bacteroides* positively correlated with increases in distance during 6MWT
Yang et al. (130)	Humans (premenopausal mostly overweight/obese Finnish women with low fitness levels)	Cycle ergometer	Cross-sectional	Composition (flow cytometry, 16S rRNA gene hybridization, DNA-staining)	VO_2max_	High VO_2max_ group had higher *Bacteroides* and lower *Eubacterium rectale*-*Clostridium coccoides* (EreC) Association between VO_2max_ and EreC disappeared after correction for fat % (association mediated by body fatness)
Yu et al. (85)	Humans (56 hypertensive Chinese adults, 65–80 years old)	Cardiopulmonary treadmill exercise test	Intervention	Composition (16S)	VO_2peak_	3 groups based on VO_2peak_: Weber A (normal exercise capacity), Weber B (mildly impaired exercise capacity), Weber C (moderately impaired exercise capacity) Lower F/B ratio in Weber A group (not statistically significant) No difference in α-diversity *Betaproteobacteria, Ruminococcaceae, Faecalibacterium* increased in Weber A group *Blautia* and *Eubacterium* increased in Weber B *Escherichia* increased in Weber C *Eubacterium* and *Blautia* positively correlated with CRP; *Alcaligenaceae* negatively correlated with CRP *Lactobacillales, Blautia, Ruminococcus*, and *E. coli* negatively correlated with VO_2peak_; *Alcaligenaceae* positively correlated with VO_2peak_

*6MWT, 6-minute walk test; DNA, deoxyribonucleic acid; EreC, Eubacterium rectale-Clostridium coccoides; F/B ratio, Firmicutes-to-Bacteroidetes ratio; PICRUSt, Phylogenetic Investigation of Communities by Reconstruction of Unobserved States; qPCR, quantitative polymerase chain reaction; rRNA, ribosomal ribonucleic acid; VO_2max_, maximal (O_2_) oxygen uptake; VO_2peak_, highest value of VO_2_ achieved during high-intensity exercise test*.

Few studies have directly investigated the effect of the gut microbiome on athletic performance (Table 4). Hsu et al. (131) and Huang et al. (132) both used germ free (GF) mice (C57BL/6JNarl) and compared these to mice colonized with bacteria to determine potential effects of the presence of the microbiome as well as specific bacteria on physical performance. Specific pathogen free (SPF) mice were found to have the highest exercise capacity and germ-free mice the lowest (131, 132). Mice colonized with individual bacterial taxa showed improvements in exercise capacity compared to their GF counterparts (131), though not all bacteria showed the same degree of impact (132). Hsu et al. (131), compared germ free (GF) mice, gnotobiotic mice colonized with *Bacteroides fragilis* (BF), and specific pathogen free (SPF) mice in a test of endurance swimming. In a similar study, Huang et al. (132) compared germ-free mice to gnotobiotic mice monocolonized with either *Eubacterium rectale, Clostridium coccoides*, or *Lactobacillus plantarum* TWK10 on performance in a swim-to-exhaustion test. In Hsu et al. (131), swim-to-exhaustion time was significantly different among all groups, with SPF mice having the greatest endurance, followed by BF mice, with GF mice having the least endurance capacity. In Huang et al. (132), gnotobiotic mice colonized with *E. rectale* showed significantly higher performance, both with and without aerobic training, than the GF mice as well as the mice colonized with *C. coccoides* and *L. plantarum*.

**Table 4:** Summary of studies investigating the effect of the gut microbiota or probiotic supplementation on exercise performance.

**References**	**Subjects**	**Type of exercise**	**Microbiota analysis/supplementation method**	**Performance metric(s)**	**Performance results**	**Host health results**
Hsu et al. (131)	Mice (C57BL/6JNarl, specific pathogen free (SPF), germ free (GF), gnotobiotic *Bacteroides fragilis* (BF); 12 wk, male)	Endurance swimming	N/A	Swim-to-exhaustion time	Swim-to-exhaustion time SPF > BF > GF Antioxidant systems glutathione peroxidase (GPx) and catalase (CAT) SPF > GF and BF; superoxide dismutase (SOD) activity SPF and GF > BF	Levels of antioxidant exyme activity GPx, SOD, and CAT: SPF > BF > GF % weight of liver, muscle, brown adipose tissue, and epididymal fat pad SPF > BF & GF
Huang et al. (132)	Mice (C57BL/6JNarl; germ free (GF), maintained germ free or colonized with *Eubacterium rectale, Clostridium coccoides*, or *Lactobacillus plantarum* TWK10; 6 wk, male)	Endurance swimming	N/A	Swim-to-exhaustion time	Swim-to-exhaustion time in specific pathogen free (SPF) > GF mice both before and after training (time increased in both SPF and GF mice after training) After training, swim-to-exhaustion time in E. rectale-colonized mice > GF, *C. coccoides*, and *L. plantarum*	*E. rectale* and *C. coccoides* mice showed higher lactate level vs. GF and *L. plantarum* Ammonia level increased more in GF group Creatine kinase (CK) lower in *E. rectale* vs. *C. coccoides* (no difference between GF and *L. plantarum*) Glucose levels higher in *E. rectale* and *C. coccoides* vs. GF and *L. plantarum* (GLUT4 higher in *E. rectale* vs. GF and *L. plantarum*) Hepatic glycogen higher in GF vs. SPF, *E. rectale, L. plantarum*, and *C. coccoides* Basal metabolic rate (BMR) higher in *L. plantarum* and *C. coccoides* mice vs. GF and *E. rectale* Wheel running distance higher in gnotobiotic mice (*E. rectale* > *L. plantarum* and *C. coccoides* > GF) Growth curve higher in GF and *E. rectale* vs. *L. plantarum* and *C. coccoides*
Chen et al. (133)	Mice (ICR, specific pathogen free (SPF), 6 wk, male)	Grip strength and endurance swimming	N/A Probiotic supplementation (*Lactobacillus plantarum* TWK10—dose-response in mice)	Grip strength and swim-to-exhaustion time	Probiotic supplementation increased grip strength and endurance swimming time after exercise	Probiotic supplementation decreased body weight, serum lactate, ammonia, urea nitrogen, albumin, CK, creatinine, triacylglycerol (TAG), and glucose and increased relative muscle weight, number of type I muscle fibers in gastrocnemius muscle
Huang et al. (134)	Humans (16 male runners)	Running (treadmill test)	N/A Probiotic supplementation (1 ×10^11^ CFU *Lactobacillus plantarum* TWK10)	Run time-to-fatigue	Probiotic supplementation increased run time-to-fatigue but not VO_2max_	Blood glucose levels higher in TWK10 group vs. placebo after exercise No significant differences in lactate, ammonia, free fatty acids (FFAs), or CK
Huang et al. (132)	Humans (54 healthy adults with no prior training; 27 men, 27 women)	Running (treadmill test)	N/A Probiotic supplementation (Lactobacillus plantarum TWK10 - placebo, low dose 3 ×10∧10 CFU, high dose 9 ×10∧10 CFU)	Run time-to-fatigue	Probiotic supplementation increased time to exhaustion in both TWK10 groups but was significantly higher in the high-dose compared to low-dose group	Lactate accumulation and ammonia production improved in the TWK10 groups during exercise and recovery phase. Blood glucose higher in high-dose group during exercise. Decrease in body fat and increase in muscle mass in high-dose group.
Jäger et al. (135)	Humans (29 recreationally-trained men)	Resistance training	N/A Probiotic supplementation (1 ×10^10^ CFU *Bacillus coagulans* GBI-30,	1 rep max (RM) one-legged leg press Vertical jump power Wingate power	Probiotic supplementation did not improve 1 RM or vertical jump power though a decrease in Wingate power was attenuated in the probiotic group	Probiotic supplementation increased perceived recovery and decreased perceived muscle soreness and measured muscle damage as indicated by CK
Lamprecht et al. (136)	Humans (23 trained men)	Cycling (cycle ergometer test)	N/A Probiotic supplementation (1 ×10^10^ CFU *Bifidobacterium bifidum* W23, *Bif*. *lactis* W51, *Enterococcus faecium* W54, *L*. *acidophilus* W22, *L. brevis* W63, *L*. *lactis* W58)	Incremental cycle ergometer exercise test	Probiotic supplementation did not improve VO_2max_ or VO_2max_ relative to body weight	Probiotic supplementation decreased zonulin and tendentially decreased carbonyl proteins and TNF-a but had no significant effects on a1-antitrypsin, malondialdehyde, total oxidation status of lipids, or IL-6
Martarelli et al. (137)	Humans (24 male cyclists)	Cycling	Plate and Randomly Amplified Polymorphic DNA (RAPD) Probiotic supplementation (1 ×10∧9 CFU/g 1:1 Lactobacillus rhamnosus IMC 501 and Lactobacillus paracasei IMC 502) vs. control	Intense exercise training	No performance results reported. Probiotic supplementation increased counts of Lactobacillus in stool (in different proportions for each subject)	Reactive oxygen metabolite (ROM) concentrations significantly increased after exercise in control group but not in probiotic group (though ROM levels not significantly different between the two groups) Biological antioxidant potential (BAP) increased after probiotic supplementation and were higher in probiotic group vs. control group
Salarkia et al. (138)	Humans (46 adolescent females)	Swimming	N/A Probiotic supplementation (4 ×10^10^ CFU/ml *Lactobacillus acidophilus* spp, *L. delbrueckii bulgaricus, Bif*. *bifidum, Streptococcus salivarus thermnophilus*) vs. ordinary yogurt	400m swim time Harvard step test	Probiotic supplementation increased VO_2max_ but did not improve 400m swim time	Probiotic supplementation reduced frequency and duration of respiratory infections and some symptoms (dyspnea and ear pain)
Shing et al. (139)	Humans (10 male runners)	Running (treadmill test)	N/A Probiotic supplementation (4.5 ×10^10^ *Lactobacillus acidophilus, L. rhamnosus, L. casei, L. plantarum, L. fermentum, Bifidobacterium lactis, Bif*. *breve, Bif*. *Bifidum, Streptococcus thermophilus*)	Run time-to-fatigue	Probiotic supplementation increased run time-to-fatigue	Probiotic supplementation reduced serum lipopolysaccharide (LPS), slightly reduced lactulose:rhamnose (gastrointestinal permeability), and gastrointestinal discomfort
Townsend et al. (140)	Humans (25 male baseball athletes)	Off-season training	N/A Probiotic supplementation (1 ×10^9^ CFU/day *Bacillus subtilis* DE111) vs. placebo	1 rep max (RM) squat and deadlift, 10 yd sprint, standing long jump	No significant differences in performance between probiotic and placebo groups	TNF-α concentrations significantly lower in probiotic group (not in any other biochemical markers) No significant differences in body composition, testosterone, cortisol, IL-10, zonulin, salivary immunoglobulin A (SIgA), and SIgM
Scheiman et al. (141)	Humans (15 athletes pre- and post- marathon, 87 ultramarathoners and olympic trial rowers pre- and post-exercise (validation cohort) vs. 10 sedentary controls)	Running	Composition (16S)	Marathon run	No performance results reported. *Veillonella* increased post-exercise in athletes	
	Mice (CL57BL/6, 12 wk, male/female?)		N/A Probiotic supplementation (*Lactobacillus bulgaricus* (control) or *Veillonella atypica* 5 ×10^9^ CFU/ml)	Run-to-exhaustion time	*Veillonella* atypica increased run-to-exhaustion time (via lactate → propionate)	Decreased inflammatory cytokines in *Veillonella*-treated mice
Soares et al. (142)	Rats (Wistar, 11 wk, male)	Running (treadmill test)	N/A Probiotic supplementation (*Saccharomyces boulardii* 1 ×10^8^ CFU/ml)	VO_2max_, run time-to-fatigue	*Saccharomyces* boulardii increased VO_2max_, run time-to-fatigue, max speed attained, and total work	Yeast supplementation had no effect on body mass gain or food intake

*BF, gnotobiotic colonized with Bacteroides fragilis; BMR, basal metabolic rate; CAT, catalase; CFU, colony forming unit; CK, creatine kinase; FFA, free fatty acid; GF, germ-free; GPx, glutathione peroxidase; ICR, Institute of Cancer Research; IL-6, interleukin-6; LPS, lipopolysaccharide; RM, rep max; SOD, superoxide dismutase; SPF, specific pathogen free; TAG, triacylglycerol; TNF-α, tumor necrosis factor alpha; VO_2max_, maximal (O_2_) oxygen uptake*.

The potential mechanisms of these effects differed between the two studies. Differences in endurance capacity in Hsu et al. were accompanied by differences in antioxidant enzyme systems, with SPF mice showing greater serum and hepatic antioxidant enzyme activity, and physiological metrics, such as weight of muscle and brown adipose tissue (131). The gut microbiome modulates adipose tissue thermogenic pathways, including browning of white adipose and activity of brown adipose, via potential mechanisms such as bile acids and the endocannabinoid system (143). The gut microbiome may also modulate skeletal muscle anabolism and function via SCFA production and alteration of the availability of intramuscular fuels (55). Mice colonized with *E. rectale* and *C. coccoides* in Huang et al. showed higher lactate levels and higher glucose levels while mice colonized with *E. rectale* showed a lower creatine kinase (CK), a marker of muscular stress, and higher wheel running distance compared to both GF mice and the other gnotobiotic mice (132).

It is worth noting, however, in Huang et al. (132) that *L. plantarum* and *C. coccoides* did not colonize stably in the mice, fecal analysis showed no significant increases of these microbes, while *E. rectale* did colonize and increase over time. Therefore, it is unclear whether the ergogenic effect was due to the presence of *E. rectale* specifically, or simply due to the successful colonization by a microbe. These studies suggest that the gut microbiome may influence performance. They also indicate that a more diverse microbiome may be more beneficial as SPF mice performed better than monocolonized BF mice (131). While Huang et al. (132) suggests that individual taxa such as *E. rectale* may be partially responsible for performance effects, further research is needed to determine exactly what aspects or taxa contribute to this ergogenic effect. These studies also did not investigate responses to a dietary or training regimen, leaving room for further research on the potential of the gut microbiome to mediate or modify exercise performance response to diet.

#### The Effect of Probiotic Supplementation on Athlete Health and Performance

While there are a number of studies on probiotic supplementation in animals and human athletes, most focus on effects such as frequency of respiratory and gastrointestinal illness or biomarkers of inflammation and immune function (137, 144–146). Supplementation of probiotic bacteria to boost the abundance or activity of potentially beneficial taxa may also serve as a potential method of modifying performance response to training. Our review of the literature found eleven studies investigating the ergogenic effect of probiotic supplementation (133–142, 147) (Table 4). Common probiotic bacteria used were strains of *Lactobacillus* or *Bifidobacterium* (133, 134, 136–139, 147). Additional strains tested included those belonging to species *Bacillus subtillis* (140) or *Bacillus coagulans* (135), *Veillonella atypica* (141), or even yeast *Saccharomyces boulardii* (142).

The majority of studies investigated the effect of probiotic supplementation on aerobic exercise performance measures such as run time-to-fatigue, VO_2max_, max speed attained, 10-yard sprint, or 400-meter swim time (132–134, 136, 138, 139, 141, 142). However, some studies also investigated strength and anaerobic outcomes such as grip strength, vertical jump power, standing long jump, Wingate power, or 1 rep max (RM) lifts (133, 135, 140).

Effects on performance variables were highly mixed between studies, though a number of studies found beneficial effects on performance parameters such as time-to-fatigue (132–134, 139, 141, 142). However, some studies found no effects of probiotic supplementation on performance metrics (136, 140) while other studies found mixed effects, with probiotic supplementation improving some performance measures, but not others (134, 135, 138). For example, Huang et al. (134) found that probiotic supplementation with *Lactobacillus plantarum* TWK10 increased run time-to-fatigue but not VO_2max_. Thus, studies reporting effects of probiotic supplementation on only one performance outcome may not be providing a complete picture of the ergogenicity of probiotic bacteria. Additionally, all but one study (137) of probiotic supplementation of humans lacked confirmation of probiotic colonization and this study acknowledged that individuals showed different levels of colonization by the probiotic bacteria. It is important that future studies investigating probiotic supplementation also collect fecal samples from participants before and after the intervention to determine whether differences in probiotic colonization may contribute to inter-individual variability in the ergogenic effect of probiotic supplementation.

In addition to performance variables, many of these studies investigated effects on body composition and inflammation. Again, results were mixed, with some studies reporting significant effects of supplementation on outcomes such as fat mass and muscle mass (132, 133) or inflammatory markers (133, 137, 139, 141, 147), though results were often mixed with some biochemical markers showing no significant effect of probiotic treatment and some studies showing no significant effects at all on these outcomes (134, 142). However, as none of these variables were analyzed, further research is necessary to determine the mechanism of the effects as well as whether the same effects are seen in humans.

#### The Effect of Antibiotic Treatment on Exercise Performance

Conversely to the use of probiotics to determine the potential effect of the gut microbiome on athletic performance, the use of antibiotics in mouse models has recently been explored to determine the potential effects of a lack of gut microbes and their metabolites on exercise capacity and muscle function (148, 149). Table 5 displays the findings of these recent studies. In both studies, antibiotic treatment decreased the exercise capacity of the mice, tested using forced treadmill running. Additionally, this phenotype could be rescued by either natural reseeding (148) or acetate infusion (149). Nay et al. also found reduced gene expression of SCFA receptor G-protein coupled receptor 41 (GPR41) and sodium/glucose cotransporter 1 (SGLT1) as well as reduced muscle glycogen in antibiotic-treated mice, suggesting that the reduced exercise capacity in these mice may have been mediated by muscle glycogen availability (148). Okamoto et al. concluded that the reduced exercise capacity of antibiotic-treated mice was due to the lack of acetate available for use as a substrate during exercise as acetyl-CoA (149). With regards to changes in the gut microbial community, Okamoto et al. reported that relative abundance of Firmicutes was increased in antibiotic-treated mice while *Bacteroidetes*, α-diversity, and fecal bacterial DNA concentration was reduced (149). Nay et al. found that fecal bacterial DNA was reduced in antibiotic-treated mice but only reported differences in composition between control mice and mice treated with antibiotics but naturally reseeded, which showed no significant differences in α- and β-diversity, *Bacteroides*, and *Firmicutes* (148).

**Table 5:** Summary of studies investigating the effect of antibiotics on exercise performance.

**References**	**Subjects**	**Type of exercise**	**Type of study**	**Diet and treatment groups**	**Microbiota method**	**Performance effects**	**Health effects**	**Microbiota effects**
Nay et al. (148)	Mice (C57BL/6J mice, 14 wk, male)	Forced treadmill running	Intervention	control (CTL) vs. antibiotics (ATB) vs. antibiotics followed by natural reseeding (NAT)	Composition and function (RT-qPCR, 16S, metagenomics)	↔ maximal aerobic velocity (MAV), extensor digitum longus (EDL) maximal strength in all groups ↓ time to exhaustion in ATB and NAT (restored in NAT after reseeding) ↓ EDL muscle fatigue index in ATB vs. CTL and NAT	↑ cecum weight in ATB vs. CTL ↓ cecum weight in NAT vs. ATB ↔ muscle mass (gastrocnemius, quadriceps, EDL soleus) in ATB vs. CTL (body weight normalized without cecum weight) ↔ myofiber phenotype, mitochondrial metabolism, inflammatory signaling, Lat1 expression, GPR40, GPR120, blood glucose ↑ Fiaf expression in ATB vs. CTL and NAT ↓ GPR41 and Sglt1 expression, muscle glycogen in ATB vs. CTL and NAT	↓ bacterial DNA in ATB and NAT (completely restored in NAT after reseeding) ↔α- and β-diversity, Bacteroides, Firmicutes between CTL and NAT
Okamoto et al. (149)	Mice (C57BL/6J mice, 10 wk, male)	Forced treadmill running	Intervention	antibiotic treatment (Abx) or antibiotic-free (Abx-free) group Acetate vs. saline infusion in Abx Butyrate infusion in Abx	Composition (16S)	↓ treadmill running time in Abx ↑ treadmill running time in Abx+acetate ↔ treadmill running time in Abx+butyrate and Abx+saline	↑ dietary intake, ceca size in Abx ↔ body mass gain, blood glucose ↓ muscle, white adipose, SCFA (fecal and plasma) in Abx ↔ body mass, muscle mass in Abx+acetate	↑ Firmicutes in Abx ↓ Bacteroidetes, diversity (Shannon), fecal bacterial DNA concentration in Abx
				Low microbiome-accessible carbohydrate (LMC) vs. high MC (HMC) diet FMT+inulin in LMC		↓ treadmill running time in LMC group ↑ treadmill running time in LMC+FMT+inulin vs. LMC	↓ muscle, fecal SCFA, plasma acetate and proprionate in LMC ↔ body mass gain, dietary intake ↑ white adipose in LMC ↔ body mass, tibialis anterior mass in LMC+FMT+inulin vs. LMC ↑ fecal SCFA in LMC+FMT+inulin vs. LMC	↑ Firmicutes, F/B ratio, Lactococcus, Allobaculum in LMC ↓ Bacteroidetes, Prevotella, S24-7, diversity (Shannon) in LMC

*Abx, antibiotic; Abx-free, non-antibiotic treated; ATB, antibiotic; CTL, control; EDL, extensor digitum longus; F/B, Firmicutes/Bacteroidetes; FMT, fecal microbiota transplant; GPR, G-protein coupled receptor; HMC, high microbiome-accessible carbohydrate; LMC, low microbiota-accessible carbohydrate; MAV, maximal aerobic velocity; NAT, antibiotic-treated and naturally reseeded; RT-qPCR, real time quantitative polymerase chain reaction; SCFA, short-chain fatty acid*.

Okamoto et al. additionally tested the effect of a low microbiota-available carbohydrate diet (LMC) vs. a high microbiota-available carbohydrate (HMC) diet to determine with substrate availability for the gut microbiome altered exercise capacity. In these treatment groups, treadmill running time was decreased in the LMC mice, concomitant with a decrease in muscle mass, fecal SCFA, and plasma acetate and proprionate as well as an increase in *Firmicutes* and decrease in *Bacteroidetes* and other SCFA producing bacterial taxa (149). This reduced exercise capacity phenotype was rescued when mice were given a fecal microbiota transplant (FMT) from HMC mice and a dose of inulin prior to exercise. The increased exercise capacity in LMC+FMT+inulin mice was not accompanied by changes in body mass or muscle mass but there was an increase in fecal SCFAs, again suggesting that SCFA concentration may act as a direct substrate or mediate substrate availability in such a way as to influence exercise capacity.

#### Summary of Findings and Putative Mechanisms of the Effect of the Gut Microbiome on Athletic Performance

**Figure 4:**
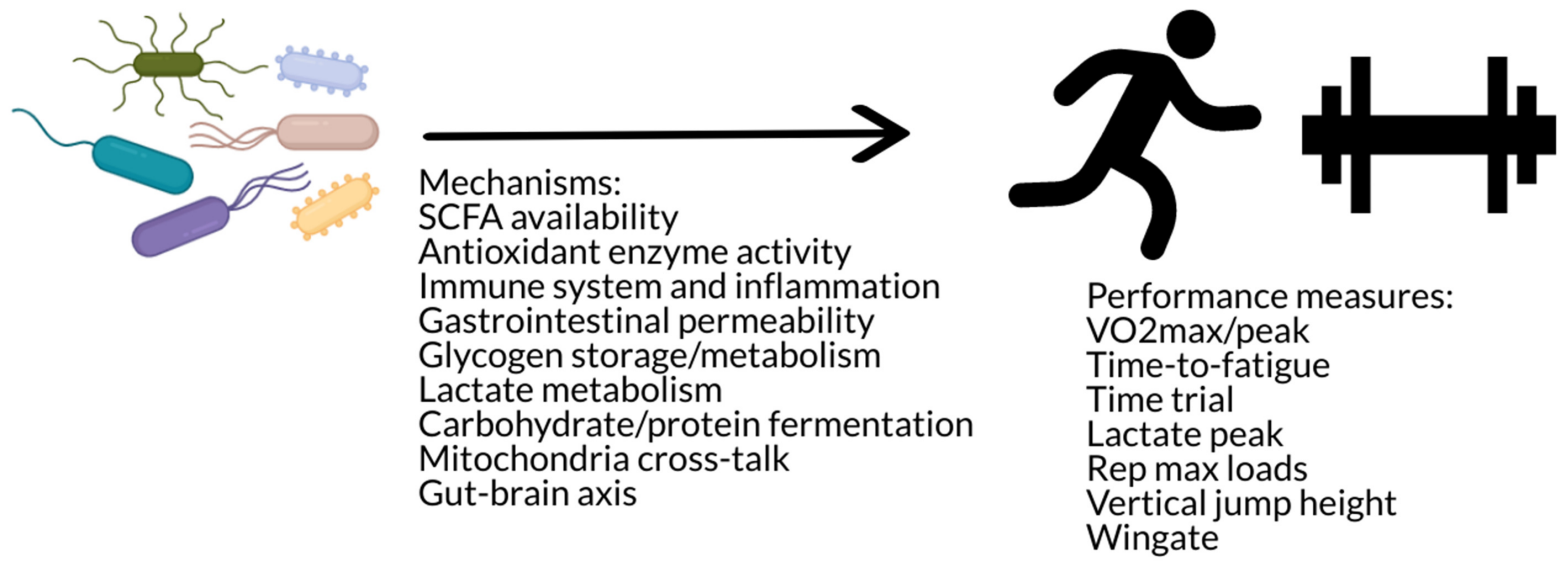
The gut microbiome may influence performance via mechanisms such as antioxidant enzyme activity, immune modulation, gastrointestinal permeability, substrate utilization and storage, mitochondria cross-talk, and/or the gut-brain axis.

These studies suggest that there may be an effect of the gut microbiome on exercise performance via mechanisms such as SCFA availability, muscle glycogen content, antioxidant enzyme activity, gastrointestinal permeability, and lactate metabolism (Figure 4). Additional speculative mechanisms may involve alterations in substrate utilization (142) as well as glycogen metabolism and storage (150), changes in neural function (142), as well as immune modulation (142) or cross-talk between the gut microbiome and mitochondria in energy production and inflammation (45). The gut microbiota has been hypothesized to impact skeletal muscle physiology and function via metabolites such as SCFAs, folate, tryptophan, glycine betaine, vitamins B_2_ and B_12_, and urolithins that may act through various pathways such as stimulation of insulin-like growth factor-1 (IGF-1), prevention of oxidative stress or inflammation, and promotion of mitochondrial biogenesis (151). However, these data also indicate that improvements in these functions do not always translate to improvements in performance. Further research is needed to investigate the effects of different probiotic strains, the interaction with dietary composition (e.g., differences of effect in athletes who have different overall dietary patterns), the use of dietary supplements, and in different modes of exercise, such as strength training.

## Conclusions and Future Directions

The gut microbiome represents an open field of study in the realm of personalized sports nutrition. High interindividual variability in response to training and physical activity is regularly reported (152) and the gut microbiome may contribute to this variability by impacting individual metabolism of food components and/or adaptation to the homeostatic stress, or training load, of the exercise stimulus (153). More research is needed to determine whether the gut microbiome could be an important predictor of athletic performance in response to dietary and exercise interventions. Researchers should refer to guides such as Ross et al. (36), Hecksteden et al. (152), Mann et al. (153), Swinton et al. (154), and Hopkins et al. (155) for statistical frameworks to interpret inter-individual variability in response and identify factors that contribute to this variability.

Specific questions that could be addressed are the role of specific bacterial taxa or groups of taxa involved in gains in athletic performance in response to certain dietary factors (e.g., protein sources such as whey, casein, soy, etc.; macronutrient distribution; or supplements such as caffeine, beta-alanine, antioxidants) or exercise stimuli. This could be investigated by using a combined dietary-exercise intervention, measuring both baseline and final microbiome and performance variables, and using predictive machine learning algorithsms such as random forests (156) to determine whether baseline abundance or changes in certain bacterial taxa can predict an individual's physical performance response. Another question is whether different taxa are involved in different responses (e.g., VO_2max_, time-to-fatigue, rep max loads, etc.) and the mechanisms of these effects (e.g., SCFA production, antioxidant enzyme activity, muscle protein synthesis, glycogen formation, energy harvest and fuel utilization, inflammation, etc.). While the first question could be addressed by a study measuring multiple exercise performance outcomes within the same population, determining the mechanisms of these effects would require *in vitro* or animal models and measurement of potential mediating metabolites, such as SCFAs, and physiological variables, such as muscle mass or muscle glycogen content. Furthermore, larger and longer studies are needed to address whether effects or responses differ between demographics (e.g., gender, age, ethnicity, etc.) and whether modulation of the gut microbiome via probiotics and/or prebiotics or modulation of the dietary or exercise stimulus (e.g., amount or type of supplement; mode, duration, intensity of exercise) may serve to increase positive response to the stimulus, decreasing the number of “non-responders.” For example, a study using the same participants and measuring microbiome and performance responses to stepwise increases in the duration and/or intensity of exercise may serve to elucidate what type of exercise may be optimal for certain individuals and their microbiomes. Additionally, studies that have measured changes in performance in response to probiotic supplementation have not looked at individual's gut microbiota composition directly. This is a limitation of these studies as different strains of probiotic bacteria show differing rates of survival through the gastrointestinal tract (157) and the composition of an individual's gut microbiota may also influence the persistence and function of probiotic bacteria in the gut (158–160). Therefore, not all probiotic strains may survive in sufficient quantities to make it down to the gut microbiome and, even if the probiotic bacteria reach the gut microbiome, it may not last as long or have the same effect in each individual.

Additional challenges and limitations in the area of research are numerous and must be taken into consideration when making claims about the exercise-microbiome connection. While the effects of diet and exercise have been shown to be orthogonal to one another (61), diet can still be a confounding factor within and between studies. Thus, conclusive effects of exercise on the gut microbiome must standardize the diet of participants, which has not yet been done. In addition to diet, variables such as genetics (32, 161), epigenetics (162), sleep behavior (163, 164), gender (165, 166), age (66, 167), and a host of other factors contribute to variability in the gut microbiome as well as performance response. This variability makes it extremely difficult to draw concrete conclusions about the effects of the gut microbiome and should always be considered when designing or interpreting studies on the interaction of the gut microbiome and host.

A related body of research has developed investigating the “gut-muscle axis” as it relates to age-related changes in muscle mass (i.e., sarcopenia) and physical frailty (151, 168–174) as well as its potential role in the “muscle-gut-brain” axis and neurodegenerative diseases in aging (175, 176). This field of research has the potential to inform the research in the field of the gut microbiome and exercise performance. Though this research focuses on preservation of muscle mass rather than physical or athletic performance, it is extremely relevant to identifying the pathways that connect these systems and how they can be modulated. Taxa, such as *Faecalibacterium prausnitzii* (151), or supplementation with prebiotics (177), butyrate (178), or other microbial metabolites such as urolithin A (179, 180) have shown beneficial associations or effects on muscle function and protection against aging-related atrophy. It has also been postulated that the aging gut microbiome may play a role in the phenomenon of anabolic resistance, not by altering protein metabolism *per se*, but by mechanisms such as gut barrier function, inflammation, and mitochondrial dysfunction (168, 170). Thus, by looking at how age-related changes in the gut microbiome may contribute to sarcopenia and decreases in muscle function, we may better understand how to modify or supplement this community to both maintain health as well as potentially increase performance.

In conclusion, there are several different fields of research that have touched on the question of the role of the gut microbiome in exercise and athletic performance. However, there are many gaps and limitations in the research thus far that must still be addressed. While there have not yet been any conclusive findings, further research and collaboration among disciplines may help shed light on the connection between exercise and the gut microbiome and the potential implications on athletic performance.

## Author Contributions

RH performed the literature review, conceived, and composed the manuscript.

### Conflict of Interest

The author declares that the research was conducted in the absence of any commercial or financial relationships that could be construed as a potential conflict of interest.
